# Family Support and Its Association with Glycemic Control in Adolescents with Type 1 Diabetes Mellitus in Riyadh, Saudi Arabia

**DOI:** 10.1155/2020/5151604

**Published:** 2020-03-23

**Authors:** Adwa M. AlHaidar, Norah A. AlShehri, Munira A. AlHussaini

**Affiliations:** ^1^Department of Family and Community Medicine, King Saud University Medical City, Riyadh, Saudi Arabia; ^2^Department of Family and Community Medicine, King Saud University, Riyadh, Saudi Arabia; ^3^College of Medicine, King Saud University, Riyadh, Saudi Arabia

## Abstract

The prevalence of type 1 diabetes mellitus (T1DM) among children in Saudi Arabia is increasing with unfavorable outcomes. Therefore, in addition to pharmacotherapy, other measures should be studied regarding psychological aspects mainly among adolescents. The family, which acts as the primary caregiver at this age, may play a major role in disease management. Thus, this study is aimed at assessing the perception of adolescents about the behaviors of their families initially and at investigating the correlation between these behaviors and glycemic control. Up to our knowledge, there was no study in Saudi Arabia that addressed this issue previously. This cross-sectional observational study assessed adolescents aged 10–19 years diagnosed with T1DM on insulin and receiving follow-up care at the King Saud University Medical City in Riyadh, Saudi Arabia. Data were collected via telephone interview and the verified “Modified Diabetes Social Support Questionnaire-Family version”. Glycemic control was then assessed using the most recent hemoglobin A1c (HbA1c) level recorded in their electronic files. Fifty-six adolescents participated in this study with an equal sex distribution (each *n* = 28). Almost all participants were Saudis, and the majority were living in Riyadh (*n* = 41). The mean age was 16.1 ± 2.41 years with a mean of 6.1 ± 4.14 years history of diabetes. The mean HbA1c level was 9.6 ± 2.12%. Participants perceived all behaviors as supportive with “support in critical situations” being the highest (77.3%) and the only factor significantly related to HbA1c (*p* = 0.017). Age was significantly related to all factors (*p* > 0.05). Family plays a major role in the management of diabetes. Their supportive behaviors are perceived by their family members diagnosed with T1DM, but there has been no optimal association with disease control. However, the involvement of the family can aid in decreasing possible complications of the disease by intervening in critical situations.

## 1. Introduction

Type 1 diabetes mellitus (T1DM) is an autoimmune disease caused by dysfunction of the beta cells of the pancreas which results in an almost absolute deficiency of insulin [1]. It is considered the most common type of diabetes among young people [2]. It is estimated that T1DM affects 1,106,200 of children and adolescents below 20 years globally. Saudi Arabia ranks eighth globally in regard to the total number of cases and the fourth in regard to incidence as it have 33.5 case per 100,000 [1].

Adolescence, a transitional stage between being a dependent child and an independent young adult, is a high-risk age for all youth. This is particularly true for those with diabetes, it is demanding on both patients and their families to adhere to multiple daily injections of insulin, glucose monitoring, and lifestyle modification. Moreover, it requires adherence to lifelong treatment [3, 4]. Reluctance to receive treatment or follow physician's advice can result in serious adverse effects, such as retinopathy, kidney disease, diabetic ketoacidosis, anxiety, and depression. This makes the family very important in the management plan.

Due to the well-known impact of psychosocial factors on patient management, specific measures, such as family support, should be taken into consideration to achieve optimal care. One study emphasized the importance of family support, as parents involved at this critical age can act as a protective factor against anxiety-related symptoms [5]. Their support is not only limited to emotional aspects but also involves guidance and supervision, encouragement of self-care and exercise, support in critical situations, and nourishment [6].

Although the element of family support and its role is limited in the literature, almost all previous studies agreed that family support has a positive association with medication adherence. A study conducted by La Greca revealed that family support in management tasks including meals, glucose testing, and insulin administration but not exercise or emotional support was associated with medication adherence. It also showed that younger adults perceived receiving more support than older ones. However, the retest reliability of the questionnaire was advised in further research [7]. Another longitudinal study supported this association as adherence to medication declines with decline in parental involvement [8]. In addition to adherence, family support predicted higher quality of life. Although the data were self-reported, the results were in accordance with the previous literature [9].

The high incidence rate of T1DM in Saudi Arabia is a burden on patients, families, and health care system either by medication itself or by complications of the disease. Further measures should be investigated to decrease the effect of T1DM and improve the role of facilities and families in the management of the disease. In the Saudi culture, family relations are dominant among other relations. In this regard, no previous study has addressed the influence of family support and the perception of patients, to our knowledge. Would Saudi Arabia show different results?

The present study was conducted to assess the perception of adolescents with T1DM about the supportive behavior of their families and to investigate the association between family support and glycemic control.

## 2. Materials and Methods

### 2.1. Study Design and Population

This cross-sectional observational study was conducted using nonprobability sampling. It included patients aged 10 to 19 years old whom are diagnosed with T1DM for at least 1 year, using insulin and following up for diabetes care at King Saud University Medical City, Riyadh, Saudi Arabia.

### 2.2. Sample Size and Recruitment Procedure

The total number of adolescents with T1DM who fulfill our criteria were sixty-three. They were all following up for diabetes care at King Saud University Medical City in different specialties (pediatrics, family medicine, endocrine, and specialized diabetes clinics). They were interviewed by trained data collectors via telephone throughout the period from April 2018 to April 2019 after their contact information was obtained from the information technology department.

### 2.3. Instruments

A telephone interview was used to collect demographic data (age, sex, nationality, residence, insulin type, duration, and chronic illnesses) in addition to the verified “Modified Diabetes Social Support Questionnaire-Family” (M-DSSQ-Family) which was adopted in this study after seeking permission [6].

The M-DSSQ-Family final model consisted of 40 items that presented a good model fit (*χ*^2^ (degrees of freedom = 686) = 964.95, comparative fit index = 0.93, and root mean square error of approximation = 0.043) with item loadings ranging from 0.40 to 0.84. Cronbach's alpha for the 40 items showed good reliabilities with the overall scale ranging from 0.92 to 0.94.

These items represented five factors. The first was “guidance and supervision” with 10 items illustrating parental behavior in advising diabetes care. Eight items described parents' encouragement on the second factor, “encouragement of self-care.” The third factor, “support in critical situations” had six items that assessed support during hyperglycemic/hypoglycemic episodes. “Nourishment,” with nine items was the fourth factor and described eating behaviors. The fifth factor assessed emotional support using seven items and was labeled “emotional support.”

The questionnaire had two parts. The first was used to rate the frequency of behaviors by answering the question “How often does one of your parents do this?” on a 6-point Likert scale with 1 = never, 2 = once a month, 3 = 1–3 times a month, 4 = once per week, 5 = several times a week, and 6 = every day. The second part assessed how adolescents rated their family members' diabetes-related behaviors for 40 items with −2 = absolutely not supportive, −1 = not supportive, 1 = neutral, 2 = supportive, and 3 = very supportive.

Then, the frequency and perceived supportiveness were multiplied to arrive at the final score. However, in our study, we only used the second part of the questionnaire in which we assessed the perceived supportiveness.

Afterward, forward and backward translation to Arabic language was performed and reviewed by a bilingual expert followed by a pilot study among 10 participants to assess the suitability and understanding of the questionnaire. This was completed easily and successfully.

### 2.4. Glycemic Control

Glycemic control was assessed using the hemoglobin A1c (HbA1c) level. The most recent HbA1c level on each patient's electronic file was recorded. HbA1c levels below 7 were considered well-controlled [1].

### 2.5. Statistical Analysis

Categorical and continuous data were described using descriptive statistics (mean, standard deviation, frequencies, and percentages). The Student's *t*-test was used to compare the mean values of independent samples. One-way analysis of variance was used to compare the mean values of quantitative outcome variables in relation to the categorical variables with more than two categories. Multivariate analysis was carried out to identify the independent variables associated with the outcome. Pearson correlation coefficient was used to measure linear correlation between two continuous variables. Statistical analyses were performed using SPSS version 21.0 (IBM Corp., Armonk, NY, USA). *p* values of ≤ 0.05 and 95% confidence intervals were used to report the statistical significance and precision of the results.

### 2.6. Ethical Consideration

The objectives of the study were explained to the family, and confidentiality of the details of the participants was ensured. Verbal informed consent was obtained from each participant and one parent before the interview. The study protocol was approved by the Institutional Review Board of the Research Unit at King Saud University Medical City prior to data collection (project no.: E-18-3112).

## 3. Results

### 3.1. Demographics

Of Sixty-three eligible participants, five participants could not be reached due to wrong telephone numbers, and the parents of two participants refused to consent to their participation. A total of 56 adolescents were interviewed with an equal sex distribution (males, 50%; females, 50%) and a mean age of 16 ± 2.41 years. The mean duration of diabetes is 6.1 ± 4.14 years. Moreover, the mean HBA1c level is 9.6 ± 2.12%. The majority of the participants are Saudis (98.2%) with 73.2% of them residing in central Saudi Arabia. Most (96.4%) participants reported using injections or pens, whereas those who used insulin pumps were quite limited in this study group (3.6%). Fortunately, 91.1% of participants denied having other chronic diseases (Table 1).

### 3.2. Factors Influencing Perceived Supportiveness

**Figure 1:**
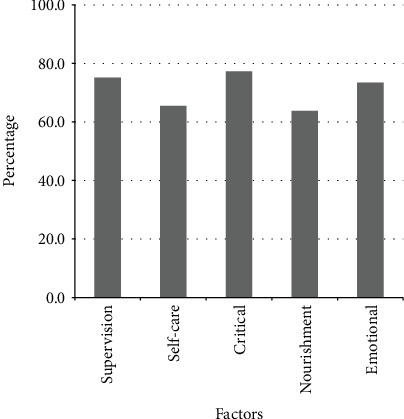
Distribution of perceived supportiveness according to the five factors.

Supervision, self-care, critical situations, nourishment, and emotional support were all perceived by adolescent as supportive behaviors; support in critical situations was most commonly reported (77.3%), followed by supervision (75.1%), emotional support (73.4%), self-care (65.5%), and nourishment (63.8%) (Figure 1). A 100% perceived supportiveness implied that the participants perceived their family behavior as extremely supportive.

### 3.3. Factors in Relation to Age

The correlation of 5 factors was assessed against the participants' ages, duration of diabetes, and HBA1c level. The Pearson correlation coefficient for linear correlation between the factors (supervision, emotional, self-care, critical, nourishment, and emotional) and numerical variables (age, duration of diabetes, and HbA1c) was assessed in this study to evaluate the relation between them (Table 2). Age had a moderate negative significant correlation with supervision (*r* = −0.471, *p* = 0.001) and critical situations (*r* = −0.417, *p* = 0.002). It also had a small negative significant relation with self-care (*r* = −0.379, *p* = 0.004), nourishment (*r* = −0.267, *p* = 0.048), and emotional (*r* = −0.285, *p* = 0.037).

### 3.4. Factors in Relation to Duration of Diabetes

The highest significant relation was between supervision and duration of diabetes (*r* = −0.647, *p* = 0.012). It was a moderate negative correlation. However, there was no significant correlation with the other factors (Table 2).

### 3.5. Factors in Relation to HbA1c

There was a small positive significant correlation between HbA1c and critical situations (*r* = 0.335, *p* = 0.017). No other relations were noticed with the remaining factors (Table 2).

### 3.6. Factors in Relation to Sex, Presence of Other Chronic Disease, and Location

Neither sex nor the presence of other chronic diseases was related to the perceived supportiveness. However, location was significantly related to supervision (*p* = 0.023), critical situations (*p* ≤ 0.001), and emotional support (*p* = 0.003) (Table 3).

## 4. Discussion

The role of the family in diabetes care, particularly T1DM, has been emphasized in the literature [10], and hence its effect on treatment adherence and glycemic control [11, 12]. Given the little number of studies that addressed the perceived supportiveness of the family behaviors, this study was carried out. These behaviors included guidance and supervision, encouragement of self-care and exercise, support in critical situations, nourishment, and emotional support.

Age has been identified as a factor related to family support. Rubin et al. suggested that as a child gets older and duration of diabetes increases, parents will be less involved in diabetes management. Moreover, Donnelly et al.'s study showed that similarly in patients with T2DM, duration of diabetes was associated with metabolic control. The shorter the duration of disease, the higher the metabolic control [13–15]. These studies were in line with our study which showed that older adolescents had lower perceived supportiveness regarding all five aspects of their family's behavior (*p* > 0.05). Longer duration of diabetes (which ultimately represented older age) was associated with lower perceived supportiveness toward supervision in particular (*p* = 0.012). As patients grow older, their autonomy develops and the feeling of dependency in diabetes care increases. This, along with their decreased perceived supportiveness, results in poor adherence and poor glycemic control.

In contrast to this, a study carried out in Brazil showed that there was a negative correlation between autonomy and age (*r* = −0.20, *p* > 0.05). However, it was a weak correlation. They attributed this to the fact that adolescents with T1DM feel less confident about being dependent and handling the responsibilities of care compared to their parents or guardians [16]. However, it is important for patients in this age group to take over the responsibility of care gradually [17]. This transition of care should be arranged in an organized manner that does not minimize the role of family support which can influence and promote health care [18].

Among all five supportive behaviors, 77.3% of the participants perceived supportiveness in critical situations. Critical situations have been described as episodes of either hypoglycemia or hyperglycemia and the family's behavior toward it such as staying with the child until he/she feels better or providing food or drinks that help to restore his blood sugar level. This high proportion of perceived supportiveness resulted in a significant but weak association between support in critical situations and HbA1c level (*p* = 0.017). On the other hand, a study conducted by Malik and Koot among Dutch adolescents revealed that “emotional support” was the only factor related to HbA1c level (*p* > 0.01) [6]. It was suggested that adolescents with higher HbA1c have uncontrolled disease and are eventually more prone to hypoglycemia and hyperglycemia [6, 19]. Thus, there is a need for rapid and urgent response from the family or caregivers, involving them more in the patients' treatment, and therefore increasing their perception. Moreover, higher HbA1c levels mandate more care and supervision to decrease the risk of hospitalization and to prevent or at least delay disease sequelae.

Regarding demographic differences, neither sex nor the presence of other chronic diseases affected the results, which can be explained by the small sample size. Since the majority of our participants lived in Riyadh (central area), they reported more support in supervision, critical situations, and emotional support compared to those who lived in other areas.

Although the participants recognized their families' behaviors as supportive, the mean HbA1c level in our sample was 9.6 ± 2.12%. Similarly, Hispanic adolescents had poor glycemic control (HbA1c = 8.9), and it was found that family support did not predict glycemic control. However, family support significantly promoted adherence (*p* > 0.01) and resulted in better glycemic control [18]. Health care system may also have a role in diabetes control. As a study conducted in Kuwait, which we share the same culture and health care system, showed that compared to Western countries, the free health care system made the family less involved in monitoring children's medical conditions and depended more on medical facilities [20].

Despite the supportive behaviors perceived by adolescents toward their families, glycemic control was not at its best. However, HbA1c is multifactorial and previous studies had investigated different factors such as “treatment adherence,” “family conflict,” and “quality of life,” which were found to be significantly correlated with it [9, 20, 21].

### 4.1. Limitations

The number of the participants was one of the limitations in the study. However, we included almost all adolescents with T1DM from KSUMC. Involvement of different centers and hospitals would have more accurate results. Moreover, the adolescents' information was self-reported so we suggest that further research would address parents' perception also. The cross-sectional type of study design is limited by specific questions whereas qualitative interview study design would generate more useful information from both the patient and his family. In addition, phone interviews cannot interpret nonverbal communication. Other variables and casual relations with metabolic control should be addressed as well. However, this research can be the first step for further research.

## 5. Conclusions

Overall, family plays a major role in the management of type 1 diabetes especially with the adolescents. The study showed that their supportive behaviors have been addressed by their diabetic family member. However, it has not been an optimal association with diabetes control. Physicians can utilize these results in promoting family support and instructing the family about its importance. They can also focus on the most supportive behavior perceived by the adolescents, and thus enhance care by practicing it more often.

## Figures and Tables

**Table 1:** Demographic data of the participants.

Demographic variable		Adolescents (%)
Sex	Male	28 (50%)
	Female	28 (50%)
Residency	Central area	41 (73.2%)
	Western area	1 (1.8%)
	Southern area	5 (8.9%)
	Northern area	8 (14.3%)
	Eastern area	1 (1.8%)
Nationality	Saudi	55 (98.2%)
	Non-Saudi	1 (1.8%)
Type of insulin	Injections/pens	54 (96.4%)
	Pump	2 (3.6%)
Presence of other chronic diseases	Yes	5 (8.9%)
	No	51 (91.1%)
Variable	Mean	SD
Age	16.1	2.41
Duration of diabetes	6.1	4.14
HbA1c level	9.6	2.12

**Table 2:** Correlation between factors and variables.

Factors	Correlation	Age	Duration of diabetes	HbA1c
Supervision	Pearson correlation	-0.471	-0.647	0.229
	Sig. (2-tailed)	0.001^∗∗∗^	0.012^∗^	0.110
	*n*	55	14	50
Self-care	Pearson correlation	-0.379	-0.399	0.193
	Sig. (2-tailed)	0.004^∗∗^	0.157	0.178
	*n*	55	14	50
Critical situations	Pearson correlation	-0.417^∗∗^	-0.279	0.335
	Sig. (2-tailed)	0.002^∗∗^	0.335	0.017^∗^
	*n*	55	14	0.017^∗^
Nourishment	Pearson correlation	-0.267	-0.323	0.005
	Sig. (2-tailed)	0.048^∗^	0.260	0.971
	*n*	55	14	50
Emotional	Pearson correlation	-0.285	0.068	0.081
	Sig. (2-tailed)	0.037^∗^	0.826	0.581
	*n*	54	13	49

^∗^ *p* > 0.05, ^∗∗^*p* > 0.005, and ^∗∗∗^*p* ≤ 0.001.

**Table 3:** Correlation between demographic data variables and factors.

Demographic variable	Factor		Adolescents (*N* = 56)	Mean	SD	*t*-test *p* value
Sex	Supervision	Male	28	68.393	15.399	0.669
		Female	28	66.786	12.451	
	Self-care	Male	28	68.527	19.187	0.212
		Female	28	62.500	16.382	
	Critical	Male	28	79.764	20.181	0.375
		Female	28	74.850	20.950	
	Nourishment	Male	28	66.364	18.974	0.251
		Female	28	61.207	13.853	
	Emotional	Male	27	74.870	14.272	0.460
		Female	28	72.061	13.746	
Location	Supervision	Central area	41	65.061	12.984	0.023^∗^
		Non-central	15	74.500	14.399	
	Self-care	Central area	41	65.168	14.931	0.814
		Non-central	15	66.458	25.013	
	Critical	Central area	41	70.227	18.644	0.001^∗∗∗^
		Non-central	15	96.660	10.713	
	Nourishment	Central area	41	61.851	15.173	0.153
		Non-central	15	69.073	19.807	
	Emotional	Central area	40	70.088	11.629	0.003^∗∗^
		Non-central	15	82.380	16.000	
Presence of other chronic diseases	Supervision	Yes	5	71.000	13.299	0.570
		No	51	67.255	14.038	
	Self-care	Yes	5	61.875	27.099	0.639
		No	51	65.870	17.137	
	Critical	Yes	5	82.500	17.785	0.558
		No	51	76.798	20.867	
	Nourishment	Yes	5	66.660	24.376	0.690
		No	51	63.504	16.032	
	Emotional	Yes	5	66.420	16.464	0.241
		No	50	74.142	13.666	

^∗^ *p* > 0.05, ^∗∗^*p* > 0.005, and ^∗∗∗^*p* ≤ 0.001.

## Data Availability

The data used to support the findings of this study are available from the corresponding author upon request.
